# Comparison of nateglinide and gliclazide in combination with metformin, for treatment of patients with Type 2 diabetes mellitus inadequately controlled on maximum doses of metformin alone

**DOI:** 10.1111/j.1464-5491.2006.01914.x

**Published:** 2006-07-01

**Authors:** S Ristic, C Collober-Maugeais, E Pecher, F Cressier

**Affiliations:** Novartis Pharma Basel, Switzerland

**Keywords:** diabetes mellitus Type 2, gliclazide, HbA_1c_, metformin, nateglinide

## Abstract

**Aims:**

To compare the effects of nateglinide plus metformin with gliclazide plus metformin on glycaemic control in patients with Type 2 diabetes.

**Methods:**

Double-blind, double-dummy, parallel group, randomized, multicentre study over 24 weeks. Patients with inadequate glucose control on maximal doses of metformin were randomized to additionally receive nateglinide (*n* = 133) or gliclazide (*n* = 129). Changes from baseline in HbA_1c_, fasting plasma glucose (FPG) and mealtime glucose and insulin excursions were examined.

**Results:**

HbA_1c_ was significantly (*P <* 0.001) decreased from baseline in both treatment groups (mean changes: nateglinide −0.41%, gliclazide −0.57%), but with no significant difference between treatments. Proportions of patients achieving a reduction of HbA_1c_ ≥ 0.5% or an end point HbA_1c_ < 7% were also similar (nateglinide 58.1%, gliclazide 60.2%). Changes from baseline in FPG were similarly significant in both treatment groups (nateglinide −0.63, gliclazide −0.82 mmol/l). Reduction from baseline in maximum postprandial glucose excursion were significant in the nateglinide group only (nateglinide −0.71, gliclazide −0.10 mmol/l; *P* = 0.037 for difference). Postprandial insulin levels were significantly higher with nateglinide compared with gliclazide. The overall rate of hypoglycaemia events was similar in the nateglinide group compared with the gliclazide group.

**Conclusions**

No significant difference was seen between nateglinide plus metformin and gliclazide plus metformin in terms of HbA_1c_. However, the nateglinide combination demonstrated better postprandial glucose control.

## Introduction

Type 2 diabetes mellitus is a progressive, chronic disease that is increasing in prevalence worldwide, along with a rise in obesity, one of its contributory factors [1]. It is characterized by insulin resistance, causing a decrease in transport of glucose into fat and muscle cells, and by a decrease in insulin secretion from the pancreas [2]. The most frequently used initial pharmacological agent is metformin, but metformin monotherapy often fails to maintain adequate glucose control over the long term. As a result of advancing deterioration of β-cell function, treatment with more than one agent is usually required over time to achieve and maintain good glycaemic control. Therefore, combining metformin with other therapies such as sulphonylureas is the next step to maintain successful control during the progressive course of the disease [3].

Sulphonylureas act by binding to sulphonylurea receptors on pancreatic β-cells, leading to increased secretion of insulin [4]. Gliclazide is a second generation sulphonylurea that is widely used in the treatment of patients with Type 2 diabetes because it has similar efficacy to other sulphonylureas but a lower risk of hypoglycaemia [5,6]. Nateglinide is a rapid-onset insulinotropic agent unrelated to sulphonylureas [7,8]. Because its effect on insulin secretion subsides when glucose levels fall, nateglinide has less potential to elicit hypoglycaemia than sulphonylureas [9]. Furthermore, nateglinide has minimal or no effect on body weight, also probably because its insulinotropic effects are limited to the postprandial period and thus nateglinide does not increase overall insulin exposure [10].

This study aimed to determine the efficacy and safety of the combination of nateglinide plus metformin with the combination of gliclazide plus metformin, in patients for whom metformin alone had failed to maintain adequate glycaemic control. Gliclazide was selected as the most appropriate comparator because of its lower risk of hypoglycaemia.

## Patients and methods

This was a double-blind, double-dummy, parallel group, randomized study carried out over a period of 24 weeks. Patients were recruited from 24 centres across five countries, as listed in the Acknowledgements. The study was carried out according to Good Clinical Practice guidelines and the Declaration of Helsinki. The protocol was approved by the appropriate ethical boards and all patients provided written informed consent. Patients were eligible if they had Type 2 diabetes for a minimum of 6 months and had received metformin monotherapy for at least 3 months; the patients also had to be on a minimum metformin dose of 1000 mg per day continuously for at least 2 months prior to study entry, but remain inadequately controlled by medication, diet and physical exercise. Other inclusion criteria were a baseline HbA_1c_ 6.8–9.0%, and a body mass index (BMI) between 20 and 35 kg/m^2^. In the 8 weeks preceding the study, and throughout the study, patients remained on their individual maximally tolerated dose of metformin.

Eligible patients received either nateglinide (Starlix, Novartis Pharma, Basel, Switzerland) or gliclazide in combination with metformin during the 24-week period. Randomization to treatment was by a computer-generated schedule via an interactive voice-responding system that assigned randomization on a study-centre basis with a block size of 4. A double-dummy technique, using identical-looking nateglinide and placebo tablets and identical-looking gliclazide and placebo capsules, was used to blind study medication assignment. Treatment regimens of nateglinide and gliclazide were started at the lowest levels (60 mg three times a day before meals and 80 mg once per day, respectively) and were titrated to the next dose level on a monthly basis up to a maximum of 180 mg before meals and 240 mg per day, respectively, during the first 3 months. Dose levels of study medication were increased if the fasting plasma glucose (FPG) level was > 7 mmol/l, if the patient had not experienced any confirmed hypoglycaemic events (symptomatic and/or asymptomatic events with plasma glucose concentration ≤ 4.0 mmol/l) and if the patient had not experienced more than three hypoglycaemic events in the past month.

Efficacy was determined from the change in HbA_1c_ levels from baseline to end point. Treatments were also assessed for the percentage of patients reaching a treatment target (end point HbA_1c_ < 7% and/or a decrease ≥ 0.5% HbA_1c_), FPG and body weight after 24 weeks of double-blind treatment. At baseline and after 24 weeks, patients attended the clinic after an overnight fast and received a standard breakfast consisting of 180 ml orange juice (grapefruit juice was not allowed), 60 g of bread (white, wheat or rye), 20 g jam or preserves, 10 g butter or margarine, 120 ml whole milk (3–4% fat) or the equivalent amount of cheese plus 120 ml water, and decaffeinated coffee or tea if desired. The meal was consumed within 15 min and blood samples were taken at 0, 15, 30, 60, 90, 120, 180 and 240 min. Glucose and insulin concentrations were measured and excursions were calculated from the difference between fasting and postprandial levels; the area under the glucose curve from 0 to 4 h (AUC_0−4 h_), adjusted for pre-meal values, was calculated for patients who had at least six measurements. In addition, insulin secretion index was calculated as: homeostatis model assessment (HOMA)-B = [20 × fasting insulin (mU/l)]/[fasting glucose (mmol/l) – 3.5].

Determinations of HbA_1c_ and glucose and insulin from the meal tests were carried out at two central laboratories, one in Europe and one in the USA. Additionally, all patients were provided with glucose-monitoring devices and supplies, and were instructed on their use. Hypoglycaemic events were analysed according to whether they were asymptomatic, symptomatic or confirmed events; number of hypoglycaemic clinical symptoms and incidence rate (number of symptoms per 100 patients per month) were also determined. Symptoms suggestive of hypoglycaemia were tachycardia, palpitations, shakiness, sweating and dizziness, hunger, blurred vision, impairment of motor function, confusion or inappropriate behaviour. An event was considered as confirmed if the self-monitored plasma glucose value obtained at the time of the event was ≤ 4.0 mmol/l (corresponding to blood glucose value of ≤ 3.6 mmol/l) or if hypoglycaemia was classified as grade 2 (i.e. an episode with sufficient neurological impairment that the patient was unable to initiate self-treatment and required assistance or hospitalization). The incidence rate of hypoglycaemic events per 100 patients per month was calculated as [(total number of events across all patients)/(total duration on treatment in days)] × 30 × 100. All adverse events were recorded; they were defined as serious if they were fatal, life-threatening, required prolonged hospitalization, resulted in persistent or significant disability/incapacity, constituted a congenital anomaly/birth defect or were considered medically significant. For each adverse event, the relationship to study drug treatment was classified by the investigator as suspected or not suspected.

A planned sample size of 120 patients per treatment was considered sufficient to detect an HbA_1c_ difference of 0.5% with 90% power, assuming a dropout rate of 15% and an sd of 1.1. An ancova model was used to test the null hypothesis that nateglinide plus metformin combination therapy was as effective as gliclazide plus metformin combination therapy. The primary ancova model included effects for treatment, study centre, baseline HbA_1c_ and treatment by baseline HbA_1c_ interaction. Efficacy analyses used the intent-to-treat population which included all randomized patients with at least one post-baseline efficacy evaluation, and safety was assessed for all randomized patients with a post-baseline safety assessment. Evaluations from the meal test were assessed for all patients with available data; during storage and transport to the central laboratories a small number of samples were lost and thus numbers of patients with available data were reduced slightly, although analysis indicated that the population with data was equivalent to the total population. The statistical tests were conducted at the two-sided significance level of 0.05.

## Results

A total of 262 subjects were enrolled in the study and their demographic details are summarized in Table 1, according to treatment group; 133 were randomized to nateglinide plus metformin and 129 to gliclazide plus metformin. The age, sex and ethnicity profiles were similar across both treatment groups. Most patients were Caucasian, with an overall mean age of 61.8 years and BMI of 29.0 kg/m^2^. Diabetic characteristics at baseline were also comparable for the two treatment groups.

**Table 1:** Demographic and background characteristics by treatment group

	Nateglinide + metformin *n* = 133	Gliclazide + metformin *n* = 129
Age (years), mean ± sd	62.0 ± 11.0	61.6 ± 10.1
Range	28–84	38–80
Age group, *n* (%)		
< 65 years	70 (52.6)	68 (52.7)
≥ 65 years	63 (47.4)	61 (47.3)
Sex, *n* (%)		
Male	72 (54.1)	65 (50.4)
Female	61 (45.9)	64 (49.6)
Race, *n* (%)		
Caucasian	131 (98.5)	124 (96.1)
Black	0	1 (0.8)
Asian/Chinese/Japanese	0	2 (1.6)
Other	2 (1.5)	2 (1.6)
BMI (kg/m^2^), mean ± sd	28.5 ± 3.5	29.5 ± 3.6
Duration of diabetes (years), mean ± sd	7.16 ± 6.30	6.70 ± 5.55
Baseline HbA_1c_ (%), mean ± sd	7.67 ± 0.59	7.60 ± 0.58
Baseline FPG (mmol/l), mean ± sd	8.95 ± 1.49	8.73 ± 1.48

During the study, 33 patients discontinued treatment prematurely; 13 (9.8%) patients from the nateglinide group (two as a result of adverse events, one lost to follow-up, nine who withdrew consent and one because of an unsatisfactory therapeutic effect) and 20 (15.5%) patients from the gliclazide group (eight due to adverse events, four lost to follow-up, six who withdrew consent and two because of protocol violations).

The mean metformin doses at study entry were comparable (1921 and 1812 mg/day in the nateglinide and gliclazide groups, respectively). The majority of patients in the nateglinide group (59.4%) were titrated up to the highest dose level (180 mg three times/day), compared with 35.7% of patients in the gliclazide group attaining the highest dose level (240 mg/day).

### Glycaemic control

HbA_1c_ was significantly decreased in both treatment groups when compared with baseline, but there was no difference in efficacy between the two treatment groups (Table 2). The proportion of patients with absolute reductions from baseline in HbA_1c_ of ≥ 0.5% were similar in both treatment groups. End point HbA_1c_ was < 7.0% in slightly fewer patients in the group treated with nateglinide than with gliclazide. However, the proportion of patients achieving either an absolute reduction of HbA_1c_ ≥ 0.5% from baseline or an end point HbA_1c_ < 7.0% was similar for both treatments.

**Table 2:** HbA_1c_ changes from baseline to end point, proportion of patients with positive responses to predefined HbA_1c_ criteria and fasting plasma glucose changes to end point

	Nateglinide + metformin *n* = 129	Gliclazide + metformin *n* = 118
Baseline HbA_1c_ (%), mean ± sd	7.66 ± 0.59	7.57 ± 0.57
Change, least squares mean ± se	−0.41 ± 0.08	−0.57 ± 0.08
* P*-value for change	< 0.001	< 0.001
Treatment difference, mean ± sd	0.17 ± 0.10	
*P*-value for treatment difference	0.099	
Response rates, *n* (%)		
Reduction of HbA_1c_ ≥ 0.5% from baseline	63 (48.8)	58 (49.2)
End point HbA_1c_ < 7.0%	45 (34.9)	55 (46.6)
Reduction of HbA_1c_ ≥ 0.5% from baseline or end point HbA_1c_ < 7.0%	75 (58.1)	71 (60.2)
Baseline FPG (mmol/l), mean ± sd	8.49 ± 1.49	8.65 ± 1.49
Change, least squares mean ± se	−0.63 ± 0.17	−0.82 ± 0.18
* P*-value for change	< 0.001	< 0.001
Treatment difference, mean ± sd	0.19 ± 0.21	
*P*-value for treatment difference	0.375	

Reductions from baseline in FPG were statistically significant in both treatment groups, but the between-group difference in change from baseline was not statistically significant (Table 2). A small increase in mean body weight of less than 0.5 kg was observed in both groups, but the changes from baseline were not significant in either case and there was no significant difference (*P* = 0.879) between treatments (data not shown).

Following the test meal, the changes from baseline to 24 weeks in maximum postprandial glucose were statistically significant for both treatment groups, but the between-group difference in change from baseline was not statistically significant (Table 3). However, the decrease from baseline in maximum postprandial glucose excursion was significant in the nateglinide group only and the decrease was significantly greater with nateglinide compared with gliclazide (*P* = 0.037). The postprandial glucose AUC_0−4 h_, adjusted for pre-meal values, was significantly decreased after 24 weeks of treatment in the nateglinide group (−2.20 mmolh/l; *P* < 0.001) but the decrease in the gliclazide group (−0.61 mmolh/l) was not significant; the difference between the changes from baseline did not reach significance (*P* = 0.054). The changes from baseline in the 30-min and 2-h postprandial insulin level and 2-h insulin excursion were larger in the nateglinide group compared with the gliclazide group. The between-group differences in change from baseline (in favour of nateglinide) were statistically significant for each parameter (Table 3).

**Table 3:** Plasma glucose and insulin levels following a test meal, and insulin secretion index (HOMA-B), at study baseline and changes after 24 weeks of treatment of patients with Type 2 diabetes

	Nateglinide + metformin	Gliclazide + metformin	Treatment difference
Maximum postprandial plasma glucose (mmol/l)	*n* = 113	*n* = 107	
Baseline, mean ± sd	15.19 ± 3.37	13.89 ± 3.08	
Least squares mean change ± SE	−1.50 ± 0.28	−1.05 ± 0.30	−0.45 ± 0.37
* P*-value	< 0.001	< 0.001	0.220
Maximum postprandial plasma glucose excursion (mmol/l)	*n* = 109	*n* = 104	
Baseline, mean ± sd	6.21 ± 3.12	5.39 ± 2.36	
Least squares mean change ± se	−0.71 ± 0.22	−0.10 ± 0.23	−0.61 (0.29)
* P*-value	0.001	0.663	0.037
30-min postprandial insulin (pmol/l)	*n* = 111	*n* = 107	
Baseline, mean ± sd	156.0 ± 118.6	156.8 ± 120.7	
Least squares mean change ± se	98.9 ± 12.1	32.5 ± 12.56	66.4 ± 15.7
* P*-value	< 0.001	0.010	< 0.001
2-h postprandial insulin (pmol/l)	*n* = 112	*n* = 100	
Baseline, mean ± sd	218.2 ± 163.6	222.6 ± 182.9	
Least squares mean change ± se	83.9 ± 16.6	39.6 ± 17.8	44.3 ± 22.2
* P*-value	< 0.001	0.028	0.047
2-h postprandial insulin excursion (pmol/l)	*n* = 107	*n* = 97	
Baseline, mean ± sd	152.2 ± 142.2	150.2 ± 166.4	
Least squares mean change ± se	75.5 ± 16.0	30.2 ± 16.6	45.3 ± 21.1
* P*-value	< 0.001	0.071	0.033
HOMA-B			
Baseline, mean ± sd	44.6 ± 48.7	56.3 ± 68.2	
Least squares mean change ± se	11.3 ± 5.3	17.3 ± 5.3	− 5.9 ± 6.9
* P*-value	0.033	0.001	0.387

*P*-values are within-group for changes from baseline and between-groups for treatment difference.

The insulin secretion index, as measured by HOMA-B (Table 3), was slightly greater at baseline in the gliclazide group than the nateglinide group, although the standard deviations were large in each case. A statistically significant increase was observed in both treatment groups after 24 weeks, but the difference between treatments was not significant.

### Safety and hypoglycaemia incidence

There were no deaths during the study. The incidence of serious adverse events, as well as of adverse events (AEs) causing dose interruption or dose change, was low and comparable between groups. Discontinuations as a result of AEs appeared to be more frequent in the gliclazide group [eight patients (6.3%)] compared with the nateglinide group [two patients (1.5%)]; for nateglinide + metformin, none of the AEs leading to discontinuation were considered related, but for gliclazide + metformin a relationship was suspected in five cases (three abdominal pain, one nausea, one dizziness/malaise). Infections and gastrointestinal disorders were the most frequently reported types of adverse events. No clinically relevant difference for any AE was noted between treatment groups. The incidence of all suspected drug-related AEs was low (6.9 and 7.1% in the nateglinide and gliclazide group, respectively).

The number of patients with at least one event suggestive of hypoglycaemia was similar between treatment groups, and the number of patients with more than one confirmed hypoglycaemic event was similar in the nateglinide group and in the gliclazide group, as shown in Table 4. The number of clinical symptoms of hypoglycaemia was nearly twice as high in the gliclazide group compared with the nateglinide group (15.5 and 28.2 symptoms per 100 patients per month in the nateglinide and gliclazide groups, respectively). In particular, fewer episodes of tremor, sweating and asthenia was reported in the nateglinide group: episodes of sweating (2.2 and 7.7 per 100 patients per month in the nateglinide and gliclazide groups, respectively), tremor (3.3 and 8.6 per 100 patients per month) and asthenia (1.2 and 5.6 per 100 patients and month).

**Table 4:** Number of patients reporting hypoglycaemic events during 24 weeks of treatment with nateglinide or gliclazide in combination with metformin

	Nateglinide + metformin *n* = 130	Gliclazide + metformin *n* = 126
Patients with at least one event suggestive of hypoglycaemia, *n* (%)	32 (24.6)	32 (25.4)
Patients with at least one confirmed event of hypoglycaemia, *n* (%)	28 (21.5)	28 (22.2)
Patients with ≥ 3 events suggestive of hypoglycaemia, *n* (%)	13 (10.0)	17 (13.5)
Patients with ≥ 3 events confirmed as hypoglycaemia, *n* (%)	12 (9.2)	16 (12.7)

## Discussion

The reduction in HbA_1c_ was similar when either nateglinide or gliclazide were added to metformin in patients who were not adequately controlled with metformin monotherapy. The degree to which the HbA_1c_ levels were lowered is in agreement with previous studies investigating the addition of nateglinide to metformin [11], or the combination of another insulin secretagogue with metformin [5,12]. It is of interest to note that, when combined with metformin, essentially equivalent efficacy was achieved with an agent targeting primarily postprandial glucose (nateglinide/metformin reduced postprandial glucose excursion to a greater extent than gliclazide/metformin) and an agent that targets primarily fasting glucose level (gliclazide/metformin reduced FPG to a greater extent than nateglinide/metformin), highlighting the importance of the postprandial period in overall glycaemic exposure [13,14].

The number of patients showing an improvement in HbA_1c_ was similar for both treatments. However, fewer patients in the nateglinide combination group achieved the target HbA_1c_ of less than 7.0% at end point than in the gliclazide group. A small difference in baseline HbA_1c_, slightly higher in nateglinide plus metformin (7.66%) compared with gliclazide plus metformin (7.57%), may account for this result. Supporting this, the composite efficacy criteria, i.e. merging an improvement of at least 0.5% or achieving an HbA_1c_ less than 7.0%, showed the treatments were comparable.

The changes in FPG concentrations mirrored those observed for HbA_1c_. Measures of post-meal glucose control and prandial insulin secretion were either significantly better in the group of patients treated with nateglinide plus metformin or showed a corresponding trend. Control of postprandial hyperglycaemia is believed to have more of an impact on both micro- and macrovascular risk factors than reduction of elevated fasting glucose levels [15]. In a meta-analysis of studies of mortality in relation to glycaemia, the 2-h blood glucose level was more predictive of all-cause and cardiovascular death than FPG [16].

Nateglinide and gliclazide were both well tolerated and the incidence of adverse events was similar in either group. The number of hypoglycaemic events, particularly asymptomatic events, was higher than seen in previous trials with nateglinide. However, this was likely to be because of the recommendation given to patients in the study to measure blood glucose regularly, independent of whether they were experiencing symptoms of hypoglycaemia. Also, the threshold value used to define hypoglycaemia was 4.0 mmol/l, which was relatively high compared with the level used routinely in many other clinical trials.

This is the first systematic comparison of the addition of a meglitinide vs. addition of a sulphonylurea to metformin as combination therapy in patients who were not adequately controlled on metformin monotherapy. A very recently published study in drug-naïve patients given either nateglinide/metformin or glibenclamide/metformin combination treatment from the start also showed similar control of HbA_1c_, but better postprandial control with nateglinide and better FPG control with glibenclamide [17]. The results of the present study showed that adding nateglinide provided similar efficacy to adding gliclazide to metformin for treatment of Type 2 diabetes, with HbA_1c_ reduced to a similar extent by either treatment over the 24 weeks. However, the nateglinide combination reduced glucose excursions and increased insulin concentrations to a greater extent than the gliclazide combination after a test meal. Addition of nateglinide to metformin is an effective treatment for patients inadequately controlled by metformin alone. Further studies are being carried out to assess long-term postprandial glucose control and relationship to diabetic complications.

## Competing interests

This study was sponsored by Novartis Pharma, Basel. The authors are employees of Novartis Pharma, Switzerland and EP has company shares. The study protocol was designed by the authors who were also responsible for data collection, analysis and reporting of results.

## Abbreviations

AEs: adverse events
AUC: area under curve
BMI: body mass index
FPG: fasting plasma glucose
HOMA: homeostatis model assessment
